# The Effect of Thiazide Diuretics on Blood Lipid Profile in Hypertensive Adults: A Meta-analysis of Randomized Controlled Trials

**DOI:** 10.7759/cureus.2651

**Published:** 2018-05-18

**Authors:** Faisal Akhtar, Faraz Khalid, Hanzhang Wang, Dongwan Zhang, Xiaolong Gong

**Affiliations:** 1 Neurology, Ochsner Health System; 2 Global Health Systems, Tulane University School of Public Health; 3 Epidemiology, Tulane University School of Public Health

**Keywords:** meta-analysis, thiazide, diuretics, metabolic markers, hypertension, lipid profile

## Abstract

This study explores the effect of diuretics use on metabolic markers (i.e., the lipid profile) since thiazide diuretics are extensively used to treat cardiac patients with hypertension (HTN) and other comorbidities.

The Embase and PubMed databases were searched for relevant English-language peer-reviewed articles. Different search terms and medical subject headings (MesH) terms were used to find the relevant articles.

Our study included randomized controlled trials with hypertensive adult patients in the intervention group receiving thiazide diuretics; controls receiving any other diuretic or any other intervention for HTN where an intention to treat analysis was performed.

We collected the demographic details, baseline lipid profile values, and end-of-study lipid profile values of all the participants in the studies along with the standard deviation of each value to calculate the net change effect.

Five studies were included. We used the Q-test and I^2^ index for heterogeneity and the inverse variance method for weighting. We used the fixed effects model for total cholesterol (TC) and low-density lipoprotein cholesterol (LDL-C) because TC and LDL-C had no heterogeneity (I^2^ was 0%). We used a random-effects model for triglycerides (TG) and high-density lipoprotein cholesterol (HDL-C), which showed moderate heterogeneity (I^2^ was 57.2% and 59.5%, respectively). We used the Cochrane quality assessment to assess the quality level of the included studies. We used a funnel plot to avoid publication bias.

Diuretics have a significant effect on lipid profiles. However, because this conclusion is supported by a low number of studies, further research is needed, and physicians are advised to use their best clinical judgment until the relationship between diuretics and lipid profiles seen in this study can be further supported by additional studies.

## Introduction and background

Hypertension (HTN) is a major cause of morbidity and mortality and an important public health challenge worldwide. Data from the National Health and Nutrition Examination Survey from 2011 to 2012 indicate the age-adjusted prevalence of HTN among adults in the United States aged 18 years and older was 29.1%. Among adults with HTN, 82.7% were aware of their HTN and 75.6% reported currently taking prescribed medication to lower their blood pressure. Worldwide prevalence estimates for HTN may be as much as one billion individuals, and complications of HTN account for 9.4 million deaths worldwide every year [1].

Among the oral medications to treat HTN, diuretics are considered one of the more effective options for the treatment of essential HTN, and their efficacy in reducing mortality in hypertensive patients has already been reported [2-3]. Diuretics were classified as the first drug of choice to start treatment in both the seventh report of the Joint National Committee on Prevention, Detection, Evaluation, and Treatment of High Blood Pressure (JNC-7) and the World Health Organization (WHO)/International Society of Hypertension Guidelines [4].

Comorbidities associated with HTN, such as diabetes and lipid disorders, are not uncommon. Previous prevalence estimates for HTN and dyslipidemia range from 15% to 31% in the United States [4]. HTN, dyslipidemia, and other comorbidities are established risk factors for many cardiovascular diseases. Given what has been previously stated, the optimal treatment for HTN should avoid worsening the patient’s metabolic profile. In some studies, diuretics, particularly thiazides, have been found to be associated with adverse effects on lipid metabolism [5-10]. Contrary to this, other studies have found the effect of diuretics on intervention and control arms to be insignificant [11-12].

Given these conflicting findings and the small scale of the studies conducted in this area, a meta-analysis evaluating the effect of thiazide diuretic treatment on lipid metabolism in hypertensive adults has been undertaken. This should allow for a more precise estimation of the intervention effect and an exploration of the heterogeneity of results from different studies.

Objective(s) and specific aims

The primary objective of this meta-analysis of randomized controlled trials (RCTs) was to examine the effect of diuretics on the blood lipid profile in hypertensive adults. For our primary analysis, we compared the effect of diuretics (thiazide) versus control groups on serum total cholesterol (TC), triglycerides (TG), high-density lipoprotein cholesterol (HDL-C), and low-density lipoprotein cholesterol (LDL-C). Secondary analyses examined whether the relationship between diuretics and the lipid profile differed by subgroups, including dosage, duration of the intervention, and type of drug.

## Review

Materials and methods

Eligibility Criteria

The studies included in this meta-analysis were RCTs conducted to assess the effects of thiazide diuretic treatment compared to other treatment regimens for controlling HTN in adults. The inclusion criteria were composed of the following key points:

1.         Patients who received thiazide diuretics were in the intervention group.

2.         Patients who received any other regimen for controlling HTN were in the comparison group.

3.         Treatment allocation was random.

4.         For parallel trials, either the net effect size or information necessary to calculate it for lipid profile differences between baseline and post-treatment times was provided. For crossover trials, post-treatment lipid levels were provided.

5.         A measure of variance, confidence interval, or P-value was provided.

6.         The age of study participants was equal to or greater than 18 years.

7.         There were no differences between comparison groups other than the specified intervention allocation.

8.         An intention to treat analysis (ITT) was performed.

Information Sources

Studies were retrieved by systematically searching the PubMed and Embase databases. PubMed was selected because it is an open-source database for clinical trials. Embase was selected because it is a biomedical and pharmacological database of published literature designed to support information managers and pharmacovigilance in complying with the regulatory requirements of a licensed drug. We searched Web of Science and ClinicalTrials.gov to avoid missing data that may be relevant to our study. A manual search of references from articles that met eligibility criteria was performed in addition to a manual review of relevant review articles, systematic reviews, and meta-analyses. Study authors were not contacted to identify additional studies.

Study Selection Criteria and Procedures

For our literature search, study selection, data abstraction, calculation of summary measures, and synthesis of results, we used a standardized written protocol; whereas for reporting results, we used the preferred reporting items for systematic reviews and meta-analyses (PRISMA) statement checklist and flow diagram.

For PubMed, medical subject headings (MeSH) terms, such as “diuretics,” “humans,” “antihypertensive agents,” “metabolism,” “lipoproteins,” and “lipids” were used as the advanced search option. Keywords like “antihypertensive,” “hypertension,” “lipids,” “metabolic,” and “randomized” were added to the search along with these MeSH terms. In addition to the aforementioned terms, synonyms and similar terms to the above were also added to the Embase search. More detailed information on our search strategy is outlined in Appendix A. All searches were filtered by RCTs and human subjects.

Two teams, each comprised of two investigators, independently reviewed articles generated by the literature search using the MeSH and keyword terms previously specified. Articles were evaluated using the inclusion criteria previously mentioned. The title and abstract of all identified articles were reviewed, and those articles deemed ineligible were excluded. Articles that met these inclusion criteria based on the initial title and abstract review were retrieved and reviewed in depth to determine further eligibility by these independent teams. For articles selected for review by both teams, the selection results were compared to ensure that all relevant articles were retrieved. A third investigator adjudicated any discrepancies between the two reviews. For the results of studies that were published more than once, only those with the most complete and up-to-date information were included in the analysis.

Data Extraction

All data were gathered using a standardized collection form. Initially, a pilot data extraction form was used; discrepancies in recorded data were discussed with all group members. As there were five members in the initial group, two sub-groups were formed for data extraction. Data were independently extracted by two researchers from each sub-group. The results from the sub-group data collection were compared. Discrepancies in the results between the two members in each sub-group were discussed to reach consensus. The characteristics of the trial and its participants were collected in the standardized data collection form. Items collected included: information for the cited trial (e.g., trial name, authors, publication year, and name of the publication), characteristics of study design, treatment period, daily dose of diuretics, and other anti-hypertensive drugs, demographics of participants (e.g., age, gender, and race/ethnicity), comorbidities, and method of statistical adjustment. For the main outcome, reviewers recorded either the mean difference in lipid profile or the mean change in lipid profile from baseline time to post-intervention, as available, corresponding standard deviations, confidence intervals, and P-values, for the absolute change in lipid profile from baseline to follow-up (mmol/L).

Quality Assessment

The quality of selected studies was assessed based on the following features:

•           Randomization

•           Blinding

•           Dropouts and withdrawals (ITT analysis)

Each of these features was included in the data extraction form.

Summary Measures

The relationship between thiazide diuretics and serum lipid level was examined by calculating the net mean change in lipid profile parameters. For parallel trials, the net mean change in lipid profile parameters was calculated by first finding the difference between trial termination (T) and baseline (B) for both the treatment (T) and control (C) arms, and then finding the difference between those: (XTT-XTB) - (XCT-XCB). For crossover trials, the net change in means was calculated by finding the mean difference in values between the end of the diuretic (trial; T) and non-diuretic (control; C) therapy periods: (XT-XC). When the average percentage of the change in lipid profile was reported by studies, the mean difference was calculated from the baseline lipid profile.

Synthesis of Results

The results of each trial were separately weighted by the inverse of the variance of change in each of lipid profile markers. If the variance for the net effect size was not reported, the variance was calculated from confidence intervals, P values, or test statistics. In parallel trials, when measures of variances were provided for control and comparison groups separately, they were pooled using the sample size within each group. For crossover studies, the variance was imputed using the paired analysis equation, assuming a correlation coefficient of 0.5 and using the variances corresponding to the mean outcome at the end of the intervention and control therapy periods. The effect estimate was then pooled by using both fixed effects and the Dersimonian and Laird random-effects models. The heterogeneity of effect size across studies was assessed by Q statistic and I2 index. Heterogeneity was explored further with an influence analysis, a sensitivity analysis, and a subgroup analysis.

**Figure 1:**
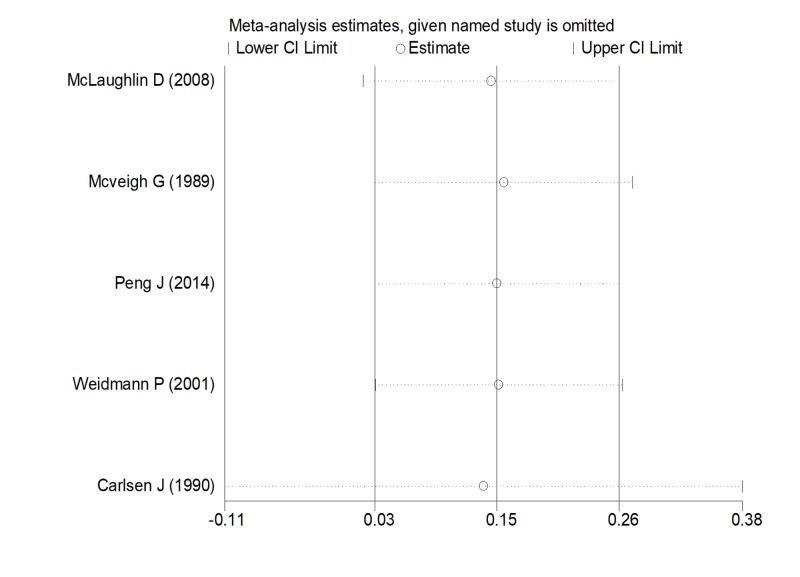
Influential analysis Abbreviation: CI, confidence interval

A sensitivity analysis was performed to assess the robustness of our findings. As part of the sensitivity analysis, we conducted a quality assessment of all studies included based on the Cochrane Handbook RevMan 5.3 user guide. Each trial was sequentially removed to determine the magnitude of its effect on the overall pooled estimate (i.e., influence analysis) (Figure 1).

**Figure 2:**
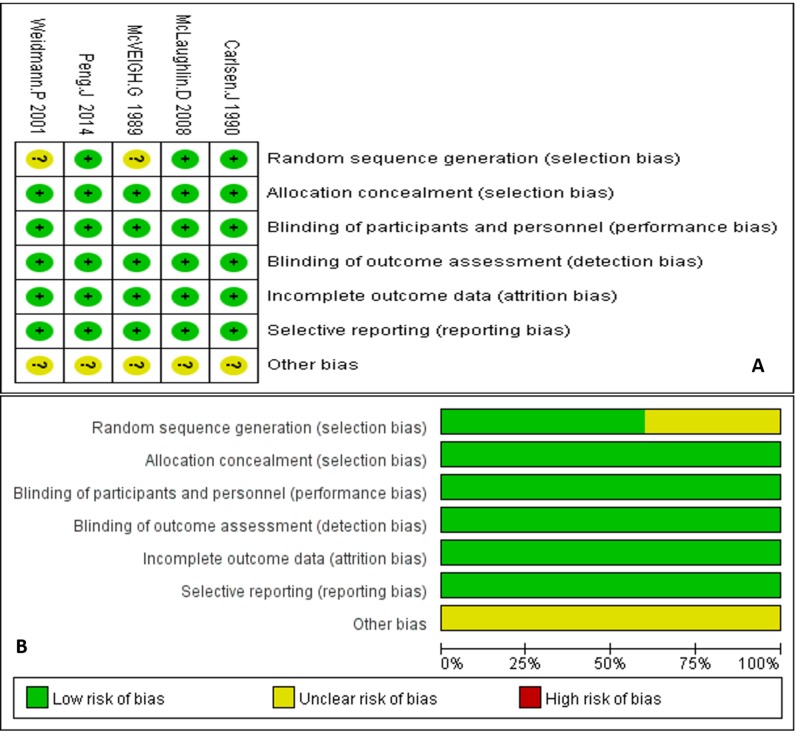
Quality assessments

Each item was judged based on the review of the full text and was labeled as either low risk (green circle), high risk (red circle), or unclear risk (yellow circle). Each circle was scored as one point (Figures 2A-2B). For example, studies that used a computer to generate random trial assignments or those studies that specifically mentioned using a collaboration center to uniformly randomize the patients were labeled as low risk for randomization of the study. For blinding, if the study mentioned blinding as either double or triple blinding, we labeled this as low risk if there was no obvious loophole to circumvent the blinding in the methods or if the article did not report an error in the blinding. For incomplete reports, we compared the patient number at baseline to that at the outcome to determine how many were lost to follow-up, drop-out, etc. Graphs were made to show the assessment of individual studies and the pooled quality assessment of our entire analysis, respectively.

The potential for publication bias was examined by a visual inspection of a funnel plot in which standard errors were plotted against the effect size for each study. The Egger regression asymmetry test was used to test the asymmetry of the funnel plot. In addition, the Begg rank correlation test was employed to examine the association between effect estimates and their variances. Analyses were conducted using Stata version 12 (Stata Corporation, College Station, Texas, USA) statistical software.

Results

Study Selection Process

**Figure 3:**
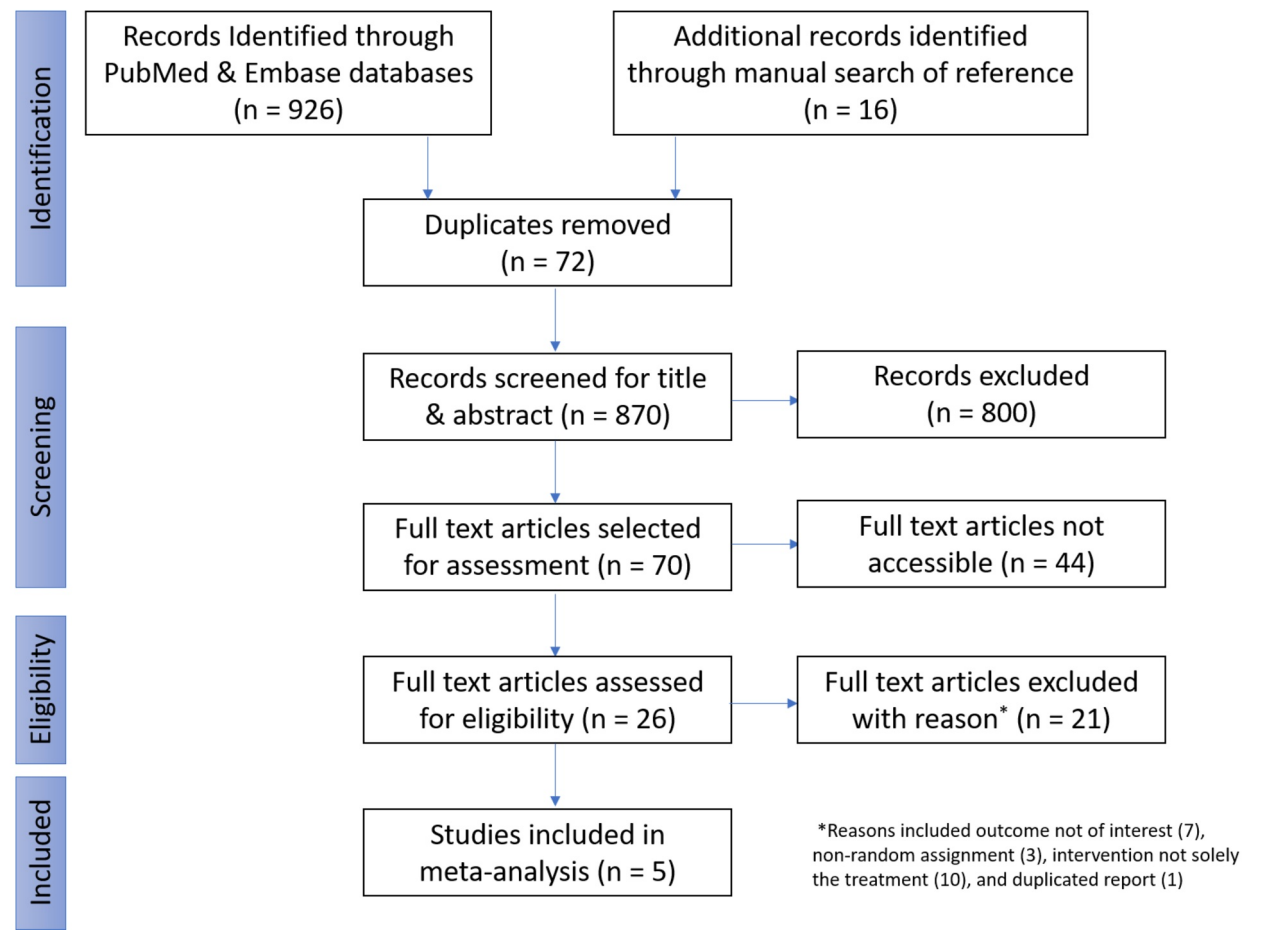
Flow diagram

In the initial phase of the selection process, searches of Embase and PubMed were performed for relevant articles that fulfill our inclusion criteria by using the previously mentioned combination of different search and MeSH terms. A total of 926 articles were selected for reviews from these databases. Duplicates were identified by using the RefWorks manager; 72 articles were found. The articles were divided between two teams of two members each, and individual independent screening of the title/abstracts of the articles was performed by both members of each team. Articles were selected or excluded on the basis of predefined inclusion criteria. Any disparity between team members was discussed and a consensus was made before finalizing the articles selected. In total, 800 articles were excluded by the two groups, and 70 articles were selected for a full-text review. Of the 70 articles, the full text was available for review for 26 while for 44 articles, the full text was not available for review. After a review of the 26 available articles, 21 articles were excluded for various reasons: on the basis of outcome (seven), non-randomized assignment (three), differences between the comparison of the intervention and the control group was not solely the treatment (10), and an additional article was found to be a duplicate (one). After exclusions, the meta-analysis of the five remaining articles was carried out (Figure 3).

Study Characteristics

We selected five blinded RCT studies that show the effect of diuretics on lipid profiles. Carlsen J et al. (1990) showed the effect of different doses of bendrofluazide on TC and TG. McVeigh G et al. (1989) showed the effect of different doses of cyclopenthiazide on TC, TG, HDL-C, and LDL-C. Weidmann P et al. (2001) demonstrated the effect of different types and doses of indapamide on TC and TG. Peng J et al. (2014) showed the effect of indapamide on TG, HDL-C, and LDL-C. A study by McLaughlin D et al. (2008) showed the effect of bendrofluazide on TC, TG, HDL-C, and LD-LC (Table 1).

**Table 1:** Study characteristics Abbreviations: C, cholesterol; HDL, high-density lipoprotein; LDL, low-density lipoprotein; SD, standard deviation; TC, total cholesterol; TG, triglyceride

Study	Year	Method	Participants	Interventions	Outcomes
Carlsen J [14]	1990	Randomized double-blind placebo-controlled parallel study	Diagnosed and treated hypertension patients	Bendrofluazide 1.25 mg Bendrofluazide 2.5 mg Bendrofluazide 5 mg Bendrofluazide 10 mg	TC, TG
McVeigh G [11]	1989	Randomized double-blind placebo-controlled parallel study	Newly diagnosed patients on monotherapy for blood pressure	Cyclopenthiazide 50 µg Cyclopenthiazide 125 µg Cyclopenthiazide 500 µg	TC, TG, HDL‑C, LDL‑C
Weidmann P [16]	2001	Randomized double-blind placebo-controlled parallel study	Patients with elevated blood pressure	Indapamide 1.5 mg Indapamide 2.5 mg	TC, TG
Peng J [12]	2014	Randomized double-blind placebo-controlled parallel study	Patients with elevated blood pressure	Indapamide 1.5 mg	TG, HDL‑C, LDL‑C
McLaughlin D [17]	2008	Randomized double-blind placebo-controlled crossover study	Patients with type 2 diabetes and elevated blood pressure	Bendrofluazide 1.25 mg	TC, TG, HDL‑C, LDL‑C

Population Characteristics

At baseline, the following population characteristics were noted (Table 2).

**Table 2:** Baseline characteristics Abbreviations: HDL, high-density lipoprotein; LDL, low-density lipoprotein; SD, standard deviation; TC, total cholesterol; TG, triglyceride

			Sex		Lipid Profile				
Author (Year)	Groups	Age in Years Mean (SD)	Male (%)	Female (%)	TC (SD) mmol/L	TG (SD) mmol/L	HDL (SD), mmol/L	LDL (SD), mmol/L	# of Participants (n = 929)
McLaughlin D (2008) [17]	Crossover	53 (2)	9 (60%)	6 (40%)	5.2 (0.7)	2.2 (0.9)	3.1 (0.2)	1.2 (0.1)	15
McVeigh G (1989) [11]	Intervention				5.9 (0.9)	1.3 (0.6)	0.9 (0.3)	4.6 (0.8)	36
	Control				5.4 (0.8)	1.3 (0.6)	0.9 (0.2)	4.1 (0.8)	11
Peng J (2014) [12]	Intervention	65.3 (7)	65 (31.1%)	144 (68.9%)		1.5 (0.5)	1.5 (0.4)	3.2 (0.9)	209
	Control	64.9 (7.1)	71 (31.3%)	156 (68.7%)		1.5 (0.4)	1.5 (0.4)	3.1 (0.9)	227
Weidmann P (2001) [16]	Intervention	55 (10)	53 (46%)	63 (54%)	6.09 (1)	1.46 (0.86)			116
	Control	53 (8)	33 (57%)	20 (43%)	6.25 (1.27)	1.57 (1.07)			58
Carlsen J (1990) [14]	Intervention	57	125 (60.98%)	80 (39.02%)	6.06 (1.12)	1.62 (1.21)			205
	Control	57	23 (44.23%)	29 (55.77%)	5.99 (0.15)	1.48 (0.17)			52

There were 45 participants in McVeigh G et al. (1989) with 36 in the intervention group and 11 in the control group. In the intervention group, the TC was 5.9 mmol/L with an SD of 0.9, TG was 1.3 mmol/L with an SD of 0.6, HDL-C was 0.9 mmol/L with an SD of 0.3, LDL-C was 4.6 mmol/L with an SD of 0.8. In the control group, the TC was 5.4 mmol/L with an SD of 0.8, TG was 1.3 mmol/L with an SD of 0.6, HDL-C was 0.9 mmol/L with an SD of 0.2, and LDL-C was 4.1 mmol/L with an SD of 0.8.

There were 257 participants in Carlsen J et al. (1990) with 205 in the intervention group and 52 in the control group. The mean age was 57 years in the intervention group, 125 (60.98%) were men, and 80 (39.02%) were women, TC was 6.06 mmol/L with an SD of 1.12, and TG was 1.62 mmol/L with an SD of 1.21. The mean age was 57 years in the control group, 23 (44.23%) were men, and 29 (55.77%) were women, TC was 5.99 mmol/L with an SD of 0.15, and TG was 1.48 mmol/L with an SD of 0.17.

There were 174 participants in Weidmann P et al. (2001) with 116 in the intervention group and 58 in the control group. The mean age in the intervention group was 55 years with an SD of 10, 53 (46%) were men, and 63 (54%) were women, TC was 6.09 mmol/L with an SD of 1, and TG was 1.46 mmol/L with an SD of 0.86. The mean age in the control group was 53 years with an SD of 8, three (57%) were men and 20 (43%) were women, TC was 6.25 mmol/L with an SD of 1.27, and TG was 1.57 mmol/L with an SD of 1.07.

There were 15 participants in the McLaughlin D et al. (2008) crossover study. The mean age was 53 years with an SD of two. Nine (60%) were men and six (40%) were women, TC was 5.2 mmol/L with an SD of 0.7, and TG was 2.2 mmol/L with an SD of 0.6, HDL-C was 3.1 mmol/L with an SD of 0.2, and LDL-C was 1.2 mmol/L with an SD of 0.1.

There were 436 participants in Peng J et al.'s study (2014) with 209 in the intervention group and 227 in the control group. The mean age was 65.3 years with an SD of seven in the intervention group, 65 (31.1%) were men and 144 (68.9%) were women, TG was 1.5 mmol/L with an SD of 0.5, HDL-C was 1.5 mmol/L with an SD of 0.4, and LDL-C was 3.2 mmol/L with an SD of 0.9. The mean age in the control group was 64.9 years with an SD of 7.1, 71 (31.3%) were men and 156 (68.7%) were women, TG was 1.5 mmol/L with an SD of 0.4, HDL-C was 1.5 mmol/L with an SD of 0.4, and LDL-C was 3.1 mmol/L with an SD of 0.9.

Statistical Analysis

The statistical results of each outcome were as follows (Table 3).

**Table 3:** Statistical analysis Abbreviations: df, degrees of freedom; ES, effect size

Outcome	I^2^ (variation in ES attributable to heterogeneity)	Heterogeneity chi-squared	Statistical Model for Analysis
Total cholesterol	0%	0.21 (df = 3) P = 0.976	Fixed effects
Triglycerides	57.2%	9.35 (df = 4) P = 0.053	Random effects
High-density lipoprotein	59.5%	4.94 (df = 2) P = 0.085	Random effects
Low-density lipoprotein	0%	1.28 (df = 2) P = 0.527	Fixed effects

Total Cholesterol

**Figure 4:**
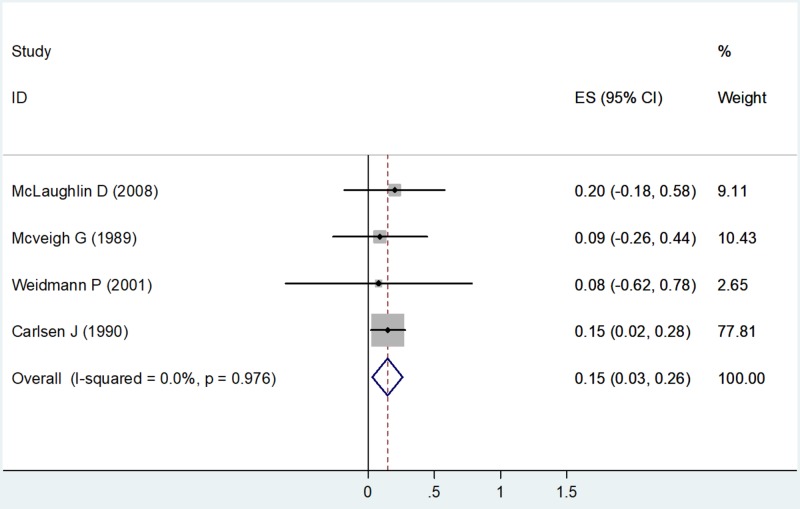
Net change in total cholesterol Abbreviations: ES, effect size; CI, confidence interval

The fixed effects model was used to calculate the effect size (ES) as the value of I2 showed 0% (no heterogeneity) with a chi-square value of 0.21 (degrees of freedom [df] = 3) and a P value = 0.976 (Table 3). Four studies had estimates for total cholesterol. Using the forest plot, the net change in TC for McLaughlin D et al. (2008) was ES = 0.20 (-0.18, 0.58), weighted 9.11. The net change in TC for McVeigh G et al. (1989) was ES = 0.09 (-0.26, 0.44), weighted 10.43. The net change in TC for Weidmann P et al. (2001) was ES = 0.08 (-0.62, 0.78), weighted 2.65. The net change in TC for Carlsen J et al. (1990) was ES = 0.15 (0.02, 0.28), weighted 77.81 (Figure 4).

Triglycerides

**Figure 5:**
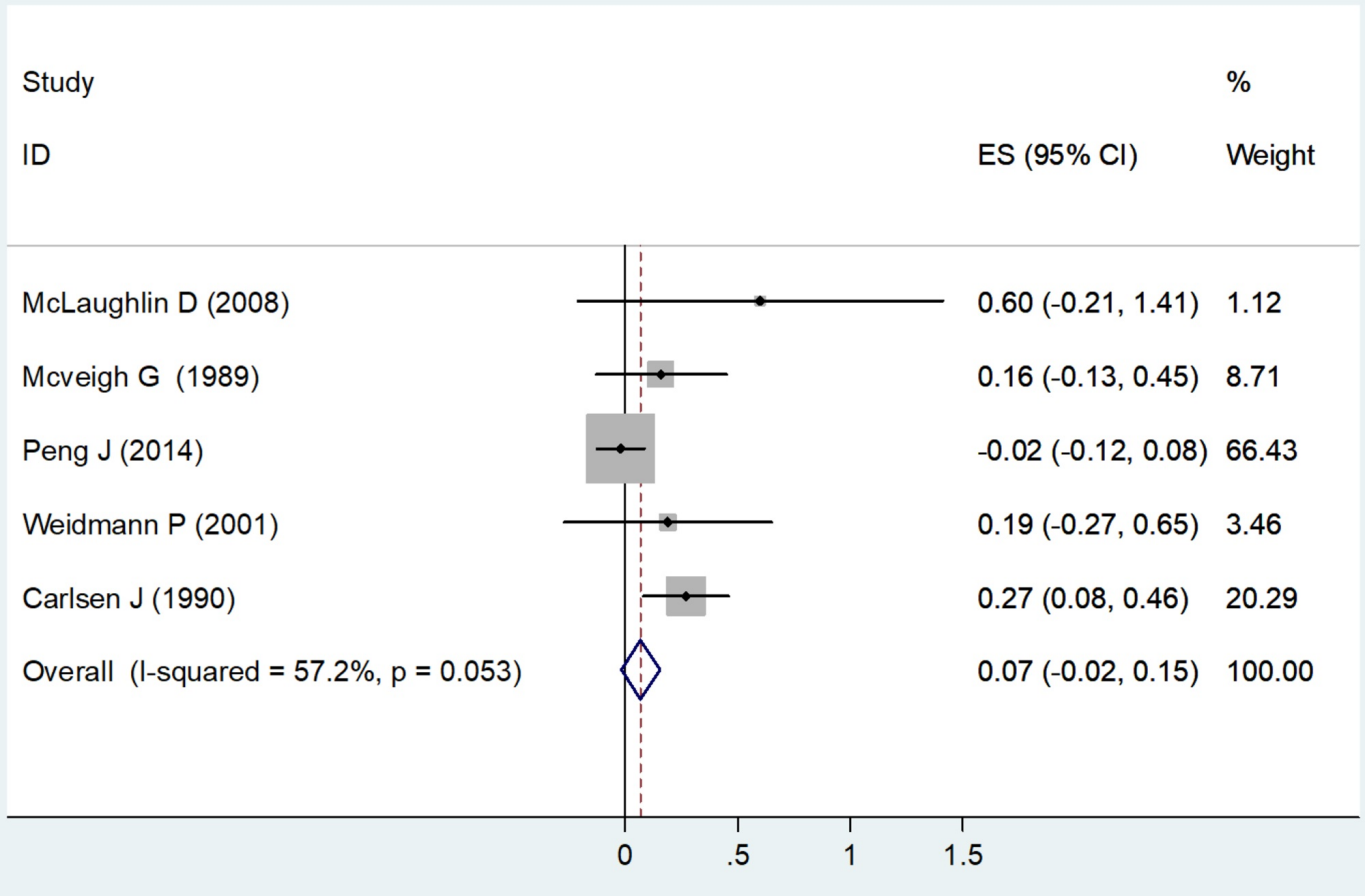
Net change in triglycerides Abbreviations: ES, effect size; CI, confidence interval

A random-effects model was used to calculate the ES as the value of I2 was 57.2% (moderate heterogeneity) with a chi-square value of 9.35 (df = 4) and P = 0.053 (Table 3). All five studies had estimates for triglycerides. Using the forest plot, the net change in TG for McLaughlin D (2008) was ES = 0.60 (-0.21, 1.41), weighted 1.12. The net change in TG for McVeigh G et al. (1989) was ES = 0.16 (-0.13, 0.45), weighted 8.71. The net change in TG for Peng J et al. (2014) was ES = -0.02 (-0.12, 0.08), weighted 66.43. The net change in TG for Weidmann P (2001) was ES = 0.19 (-0.27, 0.65), weighted 3.46. The net change in TG for Carlsen J et al. (1990) was ES = 0.27 (0.08, 0.46), weighted 20.29 (Figure 5).

High-density Lipoprotein

**Figure 6:**
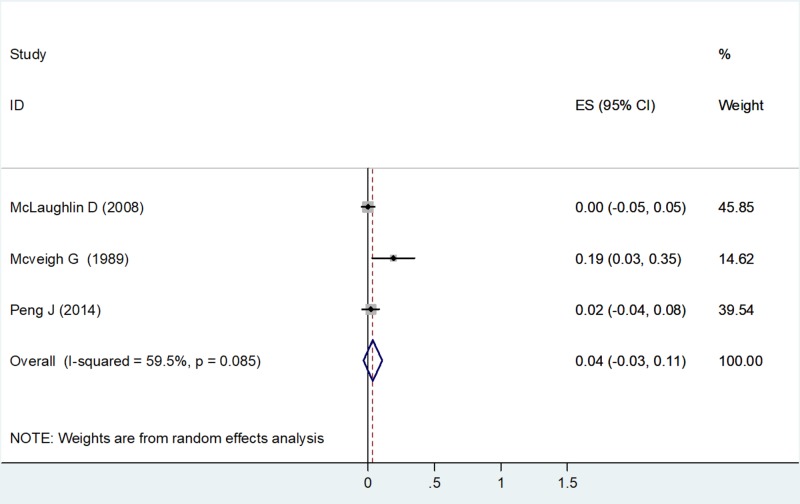
Net change in high-density lipoprotein concentrations Abbreviations: ES, effect size; CI, confidence interval

A random-effects model was used to calculate the ES, as the value of I2 was 59.5% (moderate heterogeneity) with a chi-square value of 4.94 (df=2) and a P value = 0.085 (Table 3). Three studies had estimates for HDL-C. Using the forest plot, the net change in HDL-C for McLaughlin D (2008) was ES = 0.00 (-0.05, 0.05), weighted 45.85. The net change in HDL-C for McVeigh G et al. (1989) was ES = 0.19 (0.03, 0.35), weighted 14.62. The net change in HDL-C for Peng J et al. (2014) was ES = 0.02 (-0.04, 0.08), weighted 39.54 (Figure 6).

Low-density Lipoprotein

**Figure 7:**
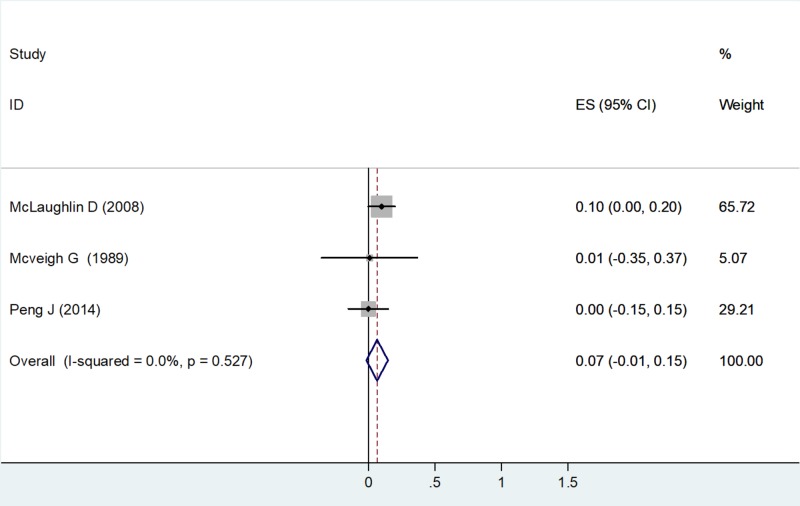
Net change in low-density lipoprotein-cholesterol Abbreviations: ES, effect size; CI, confidence interval

A fixed effects model was used to calculate the ES as the value of I2 was 0% (no heterogeneity) with a chi-square value of 1.28 (df=2) and a P value = 0.0527 (Table 3). Three studies had estimates for LDL-C. Using the forest plot, the net change in LDL-C for McLaughlin D et al. (2008) was ES = 0.10 (0.00, 0.20), weighted 65.72. The net change in LDL-C for McVeigh G et al. (1989) was ES = 0.01 (-0.35, 0.37), weighted 5.07. The net change in LDL-C for Peng J et al. (2014) was ES = 0.00 (-0.15, 0.15), weighted 29.21 (Figure 7).

Subgroup Analysis

Quality Assessment: On the basis of the Cochrane Handbook, the quality assessment of three studies showed very high results fulfilling all six quality criteria, whereas two studies fulfilled five of the six criteria in the quality assessment.

**Figure 8:**
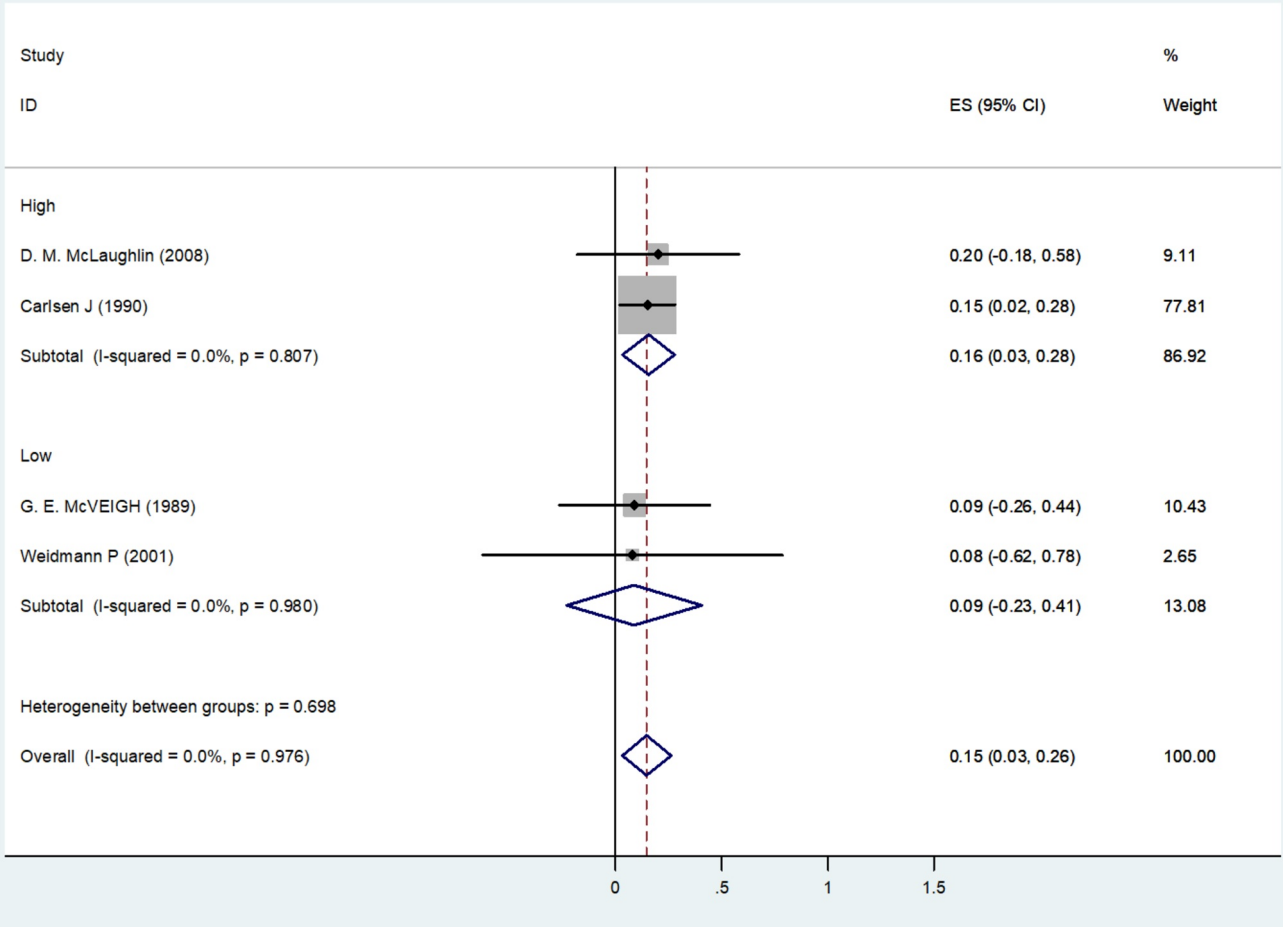
Subgroup analysis of total cholesterol based on study quality

A subgroup analysis of four studies with TC on the basis of quality assessment identified two studies with high-quality: McLaughlin et al. (2008) with an ES of 0.20 (-0.18, 0.58), weighted 9.11, and Carlsen J et al. (1990) with an ES of 0.15 (0.02, 0.28), weighted 77.81. Two studies identified as having low quality were McVeigh et al. (1989) with an ES of 0.09 (-0.26, 0.44), weighted 10.43, and Weidmann P et al. (2001) with an ES of 0.08 (-0.62, 0.78), weighted 2.65 (Figure 8).

Discussion

No meta-analysis on this topic that addresses the effects of diuretics on the lipid profile has been previously reported. The effect of any drug on metabolites in the body is of key importance to clinicians when prescribing the drug, especially if the patients are already suffering from serious conditions, including but not limited to cardiac, neurological, endocrinological, and gastrointestinal tract diseases. The selection of drugs appropriate for this group of patients is particularly important, as key drug metabolite levels can have a significant negative effect on the patient that can potentially outweigh the positive effect associated with the drug.

Many studies have shown that thiazides increase mortality and morbidity from acute myocardial infarction due to their effects on lipids profiles [13]. The antihypertensive effect of thiazides may decrease the risk of acute myocardial infarction, but this is counteracted by increases in total and low-density lipoprotein cholesterol concentrations [14]. Other studies, however, do not confirm this, with several trials finding that thiazides do not appear to alter mortality and morbidity [15].

Indapamide, a type of thiazide with a typical daily dose of 1.5 mg, has already been proven via trials, including a large number of patients, demonstrating significant results on controlling HTN efficiently without any adverse effects on metabolic markers such as serum lipids and uric acid levels [16].

Cardiac patients share a large portion of health-related conditions in the US, with HTN being a key sign with associated comorbidities such as diabetes [17]. Different treatment strategies have been used in the past (single or multiple drugs regimens) with different doses and diurnal distribution but no best combination has been found. This is unsurprising considering the metabolic disturbances to insulin resistance and lipid profiles associated with available pharmacologic options [17]. In this regard, diuretics have been found to currently be one of the most effective drugs on the market for treating patients with HTN. There are many studies that highlight the importance of diuretics for the health of hypertensive patients. Unfortunately, our literature review found very few studies that specifically addressed the effects of these diuretics on metabolic markers such as the lipid profile (TC, TG, HDL, and LDL); this is of concern, as these markers play a key role in the overall prognosis of the cardiac patient.

To find out the effect of the diuretics on the lipid profile, we performed a meta-analysis. Through the studies’ data we collected and applied statistical tools, we found out that thiazide diuretics can be a drug of choice for cardiac patients with HTN.

Many studies have different types of drugs using different doses and dosing schedules; to account for this, we calculated the pooled effect. We could see there was moderate heterogeneity in two of our outcomes for TG and HDL-C while no heterogeneity was seen in TC and LDL-C, as noted by the respective I2 indexes. A fixed effects model was used for TC and HDL-C whereas a random effects model was used for TG and LDL-C; these models are relatively more useful for each marker in terms of the generalizability of the results along with reproducibility. Though the number of studies may be low, the quality of the included studies is very high as judged by the Cochrane quality assessment. Also, according to the Cochrane Handbook RevMan user guide, this level of quality is very important in terms of the reliability of the results. No publication bias was seen based on the review of the funnel plot.

Limitations

•           One potential limitation to the present meta-analysis is that, though it is significant, how important it is from a clinical standpoint is still questionable, as it is not backed by many studies.

•           Some argue that since clinical and methodological diversity always occurs in a meta-analysis, heterogeneity will exist whether or not we happen to detect it using a statistical test.

•           In one of the studies looking at the effect of cyclopenthiazide on lipid and glucose metabolism, they observed a neutral effect in the short term due to the small numbers involved in the study. In order to determine the long-term beneficial antihypertensive activity and possible adverse metabolic effects of low dose cyclopenthiazide therapy, a larger study would be required [11].

## Conclusions

Our meta-analysis provides a good reference for clinical practice and can be helpful when considering thiazide diuretics to be prescribed to hypertensive patients with deranged metabolic markers. Our meta-analysis of the topic showed that there is a significant effect of thiazide diuretics on lipid profiles. Keeping the number of studies included in the meta-analysis in mind and the subtle change in the values observed for the lipid markers, the clinical relevance of this meta-analysis needs to be backed by an extensive literature search.

## Appendices

Search terms:

PubMed

The following search terms were used to identify relevant articles in PubMed:

Search details for intervention: Diuretics [Mesh] OR Diuretics [Pharmacological Action] OR Diuretics [Title/Abstract]) OR Diuretic [Title/Abstract]

Search details for control: Antihypertensive [Title/Abstract] OR Anti-Hypertensives [Title/Abstract] OR Antihypertensives [Title/Abstract] OR Anti-Hypertensives [Title/Abstract] OR Anti Hypertensives [Title/Abstract] OR Anti-Hypertensive [Title/Abstract] OR "Antihypertensive Agents" [Mesh] OR "Antihypertensive Agents" [Pharmacological Action]

Search details for outcome: Metabolic effects [Title/Abstract] OR metabolism [Title/Abstract] OR metabolic [Title/Abstract] OR metabolic disorder [Title/Abstract] OR metabolic abnormality [Title/Abstract] OR metabolic abnormalities [Title/Abstract] OR metabolic [Title/Abstract] OR lipids [MeSH] OR cholesterol [MeSH] OR cholesterol, hdl [MeSH] OR cholesterol, ldl [MeSH] OR triglycerides [MeSH] OR lipid [Title/Abstract] OR lipids [Title/Abstract] OR total cholesterol [Title/Abstract] OR cholesterol [Title/Abstract] OR triglyceride [Title/Abstract] OR high density lipoproteFin [Title/Abstract] OR hdl [Title/Abstract] OR low density lipoprotein [Title/Abstract] OR ldl [Title/Abstract]

Search details for limitations: humans [MeSH Terms] AND randomized controlled trial [Publication Type]

Embase

The following search terms were used to identify relevant articles in Embase:

Search details for intervention: 'diuretic' OR 'diuretics' OR 'diuretic agent'/exp

Search details for control: 'antihypertensive agent'/exp OR 'antihypertensive activity'/exp OR 'antihypertensive therapy'/exp OR 'antihypertensive' OR 'antihypertensives' OR 'anti-hypertensives' OR 'anti hypertensives' OR 'anti-hypertensive'

Search details for outcome: Note that as the bulk of the retrieved articles was irrelevant, search terms for outcome were also added into search builder - 'metabolic effects' OR 'metabolism' OR 'metabolic' OR 'metabolic disorder' OR 'metabolic abnormality' OR 'metabolic abnormalities' OR 'metabolics' OR 'lipid' OR 'Total cholesterol' OR ‘TC' OR 'Triglyceride' OR ‘TG' OR 'High density lipoprotein' OR 'HDL' ‘Low density lipoprotein’ OR 'LDL'

Search details for limitations: [humans]/lim AND ([controlled clinical trial]/lim OR [randomized controlled trial]/lim)

Final expression: Intervention AND control AND outcome AND limitations
